# Theoretical Impact of Intraocular Lens Design Variations on the Accuracy of IOL Power Calculations

**DOI:** 10.3390/jcm12103404

**Published:** 2023-05-11

**Authors:** Damien Gatinel, Guillaume Debellemanière, Alain Saad, Radhika Rampat, Jacques Malet

**Affiliations:** Anterior Segment and Refractive Surgery Department, Rothschild Foundation Hospital, 25 Rue Manin, 75019 Paris, France

**Keywords:** intraocular lens design, intraocular lens constants, lens power calculation formulas, cataract surgery

## Abstract

To ascertain the theoretical impact of optical design variations of the intraocular lens (IOL) on the accuracy of IOL power formulas based on a single lens constant using a thick lens eye model. This impact was also simulated before and after optimization. We modeled 70 thick-lens pseudophakic eyes implanted with IOLs of symmetrical optical design and power comprised between 0.50 D and 35 D in 0.5-step increments. Modifications of the shape factor resulting in variations in the anterior and posterior radii of an IOL were made, keeping the central thickness and paraxial powers static. Geometry data from three IOL models were also used. Corresponding postoperative spherical equivalent (SE) were computed for different IOL powers and assimilated to a prediction error of the formula due to the sole change in optical design alone. Formula accuracy was studied before and after zeroization on a uniform and non-uniform realistic IOL power distribution. The impact of the incremental change in optic design variability depended on the IOL power. Design modifications theoretically induce an increase in the standard deviation (SD), Mean Absolute Error (MAE), and Root Mean Square (RMS) of the error. The values of these parameters reduce dramatically after zeroization. While the variations in optical design can affect refractive outcomes, especially in short eyes, the zeroization of the mean error theoretically reduces the impact of the IOL’s design and power on the accuracy of IOL power calculation.

## 1. Introduction

The recent development of IOL power calculation formulas using thick lens models has raised interest in studying the impact of IOL design and power modifications on the refractive outcomes of cataract surgery. As specialists in intraocular lens (IOL) power calculation, we frequently encounter situations where we don’t have precise knowledge about what we’re implanting in a patient’s eye. Despite our ability to optimize formulas using IOL constants to minimize potential offsets in refractive predictions, we often observe varying prediction errors when examining the performance of a specific IOL model. This discrepancy can be particularly evident when we implant high-powered IOLs, where even when calculations are performed with utmost accuracy, there may still be unexpected refractive outcomes post-surgery. Such discrepancies can likely be attributed to changes in the optic design of the IOL across the power range, which disrupts the assumptions underlying our calculations [1]. A similar phenomenon occurs with low-powered IOLs, which can also experience significant shifts in optic configuration [2]. Recently, Olsen et al. have urged the industry to increase transparency and divulge essential information pertaining to the IOL design for precise IOL power calculation [3]. 

Effective lens position (ELP) estimation is currently considered the primary source of the prediction error of IOL power formulas [4]. The definition of the effective lens position (ELP) can differ depending on the eye model used, with refractive elements that may correspond to either thin or thick lenses. When the cornea and IOL are simulated as thick lenses, the ELP corresponds to the separation between the IOL’s principal object plane and the cornea’s principal image plane [5]. In paraxial conditions, variations in the design of an IOL considered as a thick lens induce a displacement of its principal planes and therefore influence the value of the ELP. By considering the design variations of an IOL, it becomes possible to predict the specific refractive change resulting from a deviation between the achieved ELP and its intended plane in a thick lens eye model. The theoretical influence of parameters related to the optical design of a monofocal implant on the refraction in the spectacle plane has been studied [6]. However, the theoretical impact of the monofocal optical design parameters on the accuracy of an IOL power formula has yet to be studied. A formula’s prediction error (PE) is calculated as the difference between the measured and predicted postoperative spherical equivalent (SE). Differences between different models of implants can result in the induction of systematic bias, which refers to the non-null arithmetic mean of the PE. The formulas use IOL’ constants’ that help minimize any offset found in the refractive predictions. These constants are determined based on analyzing a large dataset of eyes. If the average prediction error can be zeroed out by a constant adjustment, the impact of this adjustment on the dispersion of the average error, which depends on the accuracy of the IOL power calculation formula, cannot be easily anticipated. In this study, we investigate the theoretical impact of the optical design of an intraocular implant on the accuracy of power calculation before and after zeroization of the mean prediction error. Essential information for paraxial non-toric IOL design includes: the refractive index, central optic thickness, anterior and posterior curvature radii, and haptic angulation. We have computed the refractive impact of the implantation of IOLs with a different design from the ones for which an IOL power calculation formula is perfectly optimized. This study design was intended to simplify certain calculations and identify the theoretical refractive variations specifically related to the change of the IOL front and back radii of curvature without changing its dioptric power, central thickness, haptic design, and refractive index. By holding these variables constant and ignore other factors that may affect refractive outcomes, such as patient characteristics and surgical techniques, we aim to ensure that the observed changes in accuracy are indeed due to the IOL anterior and posterior radii manipulation. Freezing all variables except the ones of interest can be useful in controlling for confounding factors which may affect the model’s outcome and extract the specific impact of the parameters of interest from the background noise. This approach is similar to the use of a partial derivative technique, which did not seem feasible here due to the relative complexity of the analytical formulas relating the refraction of a pseudophakic eye modeled as thick lenses to the optical design of the IOL. 

## 2. Materials and Methods

In this computational modeling study, we used pseudophakic modeled eyes, all implanted with a symmetrical IOL (null Coddington shape factor) whose thickness was proportional to the IOL power. For the sake of simplicity, all modeled eyes were emmetropic for the reference design at baseline. To assess the influence of the optical design of the IOL on ocular refraction for the same anatomical position of the optic, we carried out simulations by modifying the Coddington shape factor or optical design of IOLs while keeping their anatomical distance and thickness unchanged. The refractive outcomes resulting from changes in intraocular lens (IOL) design can be determined using Equation (A1) (Appendix A), which calculates the refraction for a thick lens pseudophakic eye, taking into account various biometric factors such as ELP. In this situation, the computed spherical equivalent (SE) equals the refractive variation and can be equated to the fraction of the formula prediction error caused by the alteration of the ELP due to the design change. The prediction error analysis was then performed using mean absolute errors, standard deviation, and RMS error [7].

### 2.1. Prediction Error (PE), Mean PE, Mean Absolute Error, and Standard Deviation of the PE

For a given eye, the prediction error E_j_ of an implant power calculation formula is equal to the difference between the achieved and predicted SE:

(1) $$E_{j}={SE}_{a}-{SE}_{p}$$

The mean prediction error of a formula on a set of N eyes is equal to:

(2) $$\overset{-}{E}=\frac{1}{N}\sum_{j=1}^{N}E_{j}$$

The mean absolute error (MAE) is calculated as:

(3) $$MAE=\frac{1}{N}\sum_{j=1}^{N}{|E}_{j}|$$

The standard deviation (SD) of the error (σ) is given by:

(4) $$\sigma^{2}=\frac{1}{N}\sum_{j=1}^{N}{\left(E_{j}-\overset{-}{E}\right)}^{2}$$

When ($\overset{-}{E}\neq 0$), the lens constant can be adjusted to achieve the zeroization of the mean PE. This method leads to determining the value of a constant increment, added to each IOL’s predicted ELP (ΔELP), so that the refractive change produced (ΔE_j_) is such that:

(5) $$\sum_{j=1}^{N}\left(E_{j}+{∆E}_{j}\right)=0$$

After zeroization, the MAE becomes:

(6) $$MAE=\frac{1}{N}\sum_{j=1}^{N}{|E}_{j}+{∆E}_{j}|$$

The standard deviation σ is given by:

(7) $$\sigma^{2}=\frac{1}{N}\sum_{j=1}^{N}{\left(E_{j}+{∆E}_{j}\right)}^{2}$$

### 2.2. Uniform vs. Non-Uniform IOL Power Distribution

Suppose n_p_ is the number of IOLs of the same power P within a distribution of N implants. In that case, the distribution is uniform if n_p_ is constant regardless of P, and not uniform otherwise. The frequency of a class of implants with the same power P equals n_p_/N.

To isolate the impact of the design of the optics of an IOL, the biometric parameters were kept constant for the same class of implant P. 

If $\overset{-}{E_{p}}$ the average error made on a set of eyes that received the same IOL, then

(8) $$\overset{-}{E}=\sum_{\begin{array}{c} p\;=\;min\;to\;max\\ by\;0.5\;steps \end{array}}\frac{n_{p}}{N}{\overset{-}{E}}_{p}$$

(9) $$MAE=\sum_{\begin{array}{c} p\;=\;min\;to\;max\\ by\;0.5\;steps \end{array}}\frac{n_{p}}{N}{\overset{-}{|E}}_{p}|$$

(10) $$\sigma^{2}=\frac{1}{N}\sum_{\begin{array}{c} p\;=\;min\;to\;max\\ by\;0.5\;steps \end{array}}^{N}n_{p}{\left({\overset{-}{E}}_{p}-\overset{-}{E}\right)}^{2}$$

Zeroization can be achieved by adding a constant increment to each IOL. This increment (ΔELP) will be the same for every IOL of power P and produce a refractive change (ΔE_p_) such that:

(11) $$MAE=\frac{1}{N}\sum_{\begin{array}{c} p\;=\;min\;to\;max\\ by\;0.5\;steps \end{array}}^{N}n_{p}|{\overset{-}{E}}_{p}+\Delta E_{p}|$$

(12) $$\sigma^{2}=\frac{1}{N}\sum_{\begin{array}{c} p\;=\;min\;to\;max\\ by\;0.5\;steps \end{array}}^{N}n_{p}{\left({\overset{-}{E}}_{p}+\Delta E_{p}\right)}^{2}$$

(13) $$RMS={\left(\sigma^{2}+{\overset{-}{E}}^{2}\right)}^{0.5}$$

### 2.3. IOL Optic Design

In this study, the design of an implant considered a thick lens allows the distribution of its optical power between its front and back. 

The Coddington shape factor (CF) is a formal measure of the bending of a lens. It is calculated using the usual sign convention for radii of curvatures of a lens’s anterior and posterior surfaces.

The CF is given by:

(14) $$X=\frac{\left(R_{ip}+R_{ia}\right)}{\left(R_{ip}-R_{ia}\right)}$$

It is null for a symmetrical biconvex or biconcave lens. It equals −1 and 1 for planoconvex lenses (for a flat lens surface located anteriorly and posteriorly, respectively). It is less than −1 or more than 1 for meniscus lenses, depending on the sign of the radii and their relative absolute value. The bending influences the location of the principal plane compared to the lens surfaces. Thus, by keeping all parameters constant except the lens’s optical design (same anatomical distance, same thickness), we can observe variations in the refraction of the pseudophakic eye induced by the sole change in IOL design.

Most companies that manufacture these implants do not reveal the design data but simply the nominal power of the implants and their refractive index. However, we obtained data on the geometry of certain models of intraocular lenses from their manufacturers and utilized them in our simulations.

### 2.4. Refractive Impact of IOL Design

As in a previous paper [8], we used a four refractive surfaces, thick paraxial pseudophakic eye model. To study the specific impact of the change in IOL design, we modeled 70 emmetropic pseudophakic eyes implanted with a reference IOL of symmetrical design (null CF) and power comprised between 0.5 D and 35 D with 0.50 D incremental steps. 

We then built two sets: the first set corresponds to a uniform IOL power distribution (containing the same number of IOLs for each of the 70 different powers). In contrast, the distribution of the second set was intended to reflect that of the IOL’s power in a general population of pseudophakic eyes. 

Then, the CF value was modified for all dataset’s IOLs, without changing the anatomical position (distance between the corneal vertex to the IOL vertex) or the IOLs’ thickness. We performed numerical simulations to calculate the SE refraction (ΔR) change resulting from a given thick lens design variation for each IOL power. We assimilated this refractive variation to the specific contribution of the variation in IOL’s optical design to the prediction error of the calculation formula. We computed the mean PE, the standard deviation (SD) of the PE, the Mean absolute error, the RMS, and the maximum absolute induced PE before and after zeroization of the mean P for each distribution. The refractive variations obtained after zeroing out the mean PE, were also calculated. Zeroing out the mean PE was performed by adding an increment in ELP (ΔELP), which was determined analytically, to the baseline ELP value.

The details of these calculations are presented in Appendix A.

The study adhered to the tenets of the Declaration of Helsinki and was approved by the Rothschild Foundation Hospital institutional board (IRB 00012801) under the number CE_20211123_6_GDE.

## 3. Results

We modeled 70 pseudophakic eyes having the same total corneal power (Rca = 7.7 mm, Rcp = 6.8 mm, t = 0.535 mm, K = 43.06 D using Equation (A2), where each received the same symmetrical biconvex IOL (null Coddington shape factor) with a different power ranging between 0.5 D and 35 D by 0.50 D incremental steps. All modeled eyes were emmetropic for their initial ELP, corneal and IOL powers, and axial length. This configuration would correspond to the situation obtained postoperatively, owing to a “perfect” theoretical calculation formula. This methodology simplifies the model here, with the numerical value of the SE directly corresponding to the difference between the predicted refraction and the observed refraction. The refractive index values used for numerical simulations were: n_a_ = 1.336 (aqueous), n_v_ = 1.336 (vitreous), n_c_ = 1.376 (corneal stroma), and n_iol_ = 1.5 (IOL material). The distance to the spectacle plane was set to d = 12 mm. The biometric parameters of these modeled eyes and symmetrical IOLs design characteristics are reported in Table 1.

### 3.1. Design of the Non-Uniform IOL Power Distribution

For the non-uniform distribution, we used a dataset comprising 20,872 eyes undergoing cataract surgery at our institution. These eyes did not have any previous ocular surgery. The data were anonymized and contained preoperative biometric data derived from the IOL-Master 700 (Carl-Zeiss-Meditec, Jena, Germany), including axial length AL, anterior chamber depth ACD (from front corneal apex to the front apex of the crystalline lens), lens thickness LT, and the front corneal surface flat (R_ca_) and steep (R_cp_) radii. We used the PEARL DGS formula [9] for each eye to calculate the theoretical emmetropizing IOL power. After applying the three-point moving mean, we rounded it to the nearest 0.5 dioptric power. We used an equivalent A constant of 118.9. We obtained a unimodal frequency (n_p_/N) histogram representative of the distribution of IOL powers necessary to induce emmetropia in our general population, presented in Appendix B.

### 3.2. Impact of the Coddington Shape Factor before and after Zeroing out the Mean PE

We varied the Coddington shape factor of each IOL of uniform and non-uniform distributions by ±0.1 increments between −1 and +1 without modifying any other parameters (same IOL thickness and anatomical distance to the corneal front surface). 

**Figure 1:**
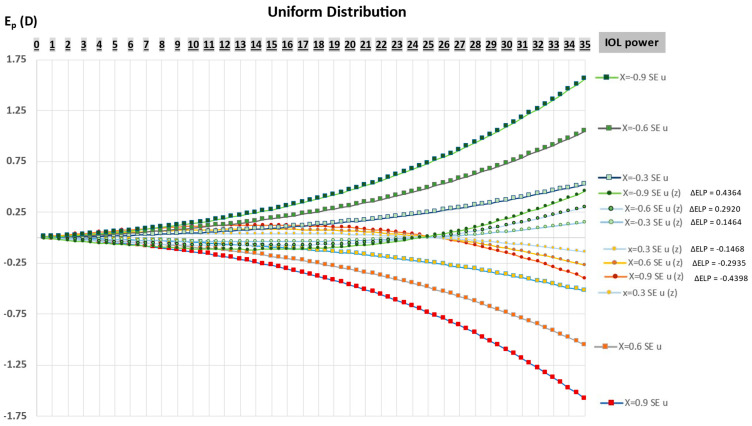
Spherical equivalent (SE = E_p_) as a function of the change in the IOL design and power in a uniform (u) distribution (n_p_ = 1) before and after zeroization with an ELP increment (ΔELP) for discrete variations of the Coddington factor. Before zeroization, a positive departure of the CF from 0 causes a negative SE, whereas a negative departure of the CF causes a positive SE. These values increase exponentially with the IOL power. After zeroization, the sign of SE depends on IOL power; the inversion occurs for all curves at a power level close to 25D.

Figure 1 represents the change in SE incurred by a departure from 0, of the Coddington shape factor of ±0.3 steps, before and after zeroization for the uniform IOL power distributions. Before zeroization, positive variation in CF would lead to myopic SEs, which increase exponentially with the IOL power. After zeroization obtained by a negative increment (delta ELP < 0), SEs become hypermetropic for powers below a value close to 25 D and remain myopic beyond. A symmetrical trend is observed for a negative variation in Coddington’s factor. Zeroization induces a clear reduction of the induced refractive variation and a tightening of the curves near the x-axis. At the highest IOL powers, the effect of zeroization is significant enough to reduce the impact of design changes to approximately one-third of its original value. 

**Figure 2:**
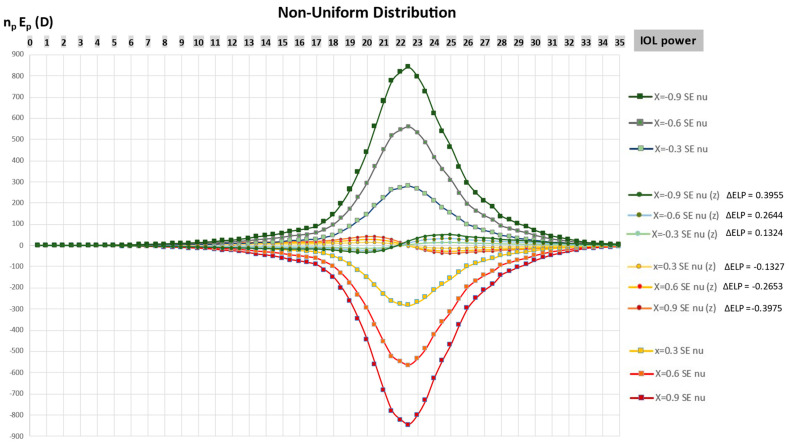
Cumulative SE variations (n_p_E_p_) as a function of the change in the IOL design and power in a non-uniform (nu) distribution before and after zeroization (z) with an ΔELP increment for discrete variations of the Coddington factor.

Figure 2 shows the cumulative SE change (n_p_E_p_) incurred by a departure from 0 of the Coddington shape factor, by ±0.3 steps, for the non-uniform unimodal distribution, before and after zeroization. Before zeroization, the peak of the cumulative SE variations r is induced by the power most frequently used in our simulation. After zeroization, there is a symmetric distribution around the horizontal axis of the cumulative refractive change where the amplitude is dramatically reduced, and the sign reverses beyond an IOL power close to the most represented value. 

**Figure 3:**
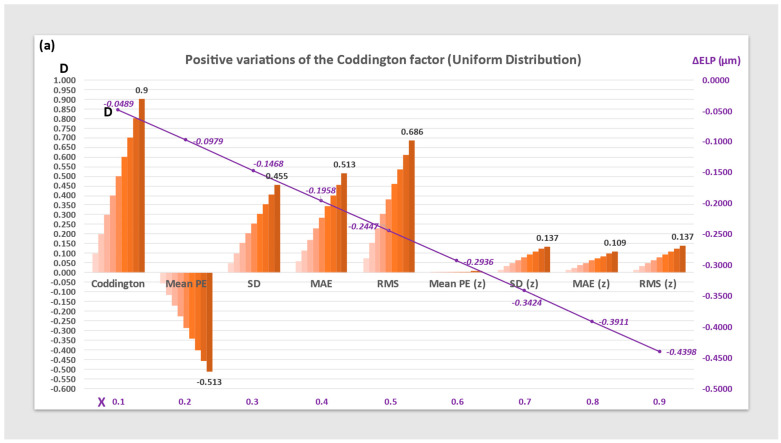

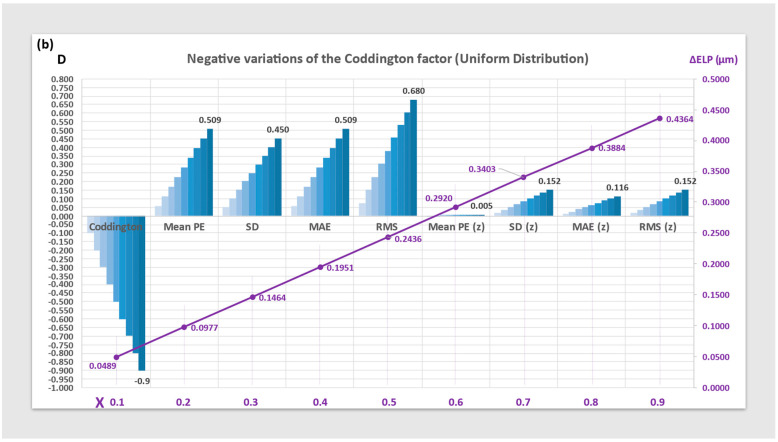
Histograms of the Mean prediction error (Mean PE), Standard Deviation (SD), Mean Absolute Error (MAE), and Root Mean Square (RMS) of the PE for positive (**a**) and negative (**b**) variations of the Coddington shape factor before and after zeroization (z) using an ELP increment (ΔELP) on a uniform distribution. The maximum values obtained for each parameter are shown on the graph (Diopters, left ordinate axis). The value of the ELP increment used for zeroization is plotted against the CF value (in mm, right ordinate axis).

Figure 3 represents the variations of the uniform distribution’s Mean PE, SD, MAE and, RMS before and after zeroization using a constant increment (ΔELP).

All represented parameters increase linearly with the value of the Coddington factor. Their value is significantly reduced after zeroization. ELP increments required for zeroization have an opposite sign and magnitude proportional to the value of the Coddington factor.

**Figure 4:**
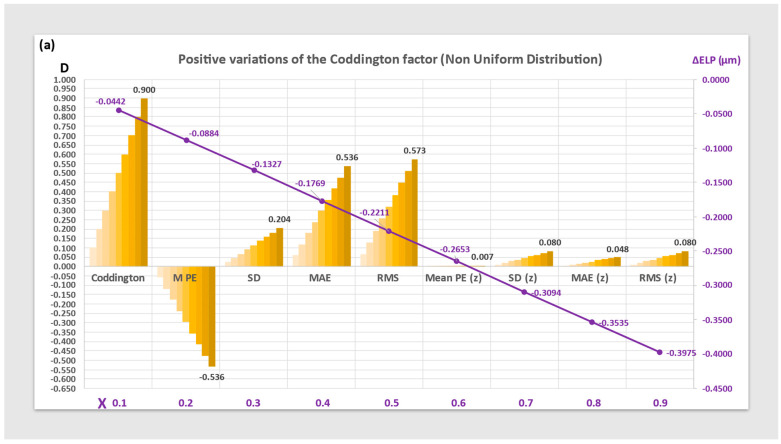

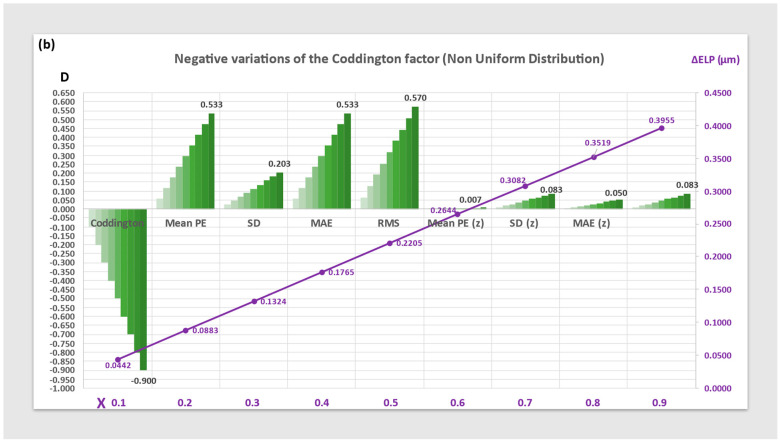
Histograms of the Mean prediction error (Mean PE), Standard Deviation (SD), Mean Absolute Error (MAE), and Root Mean Square (RMS) of the PE for positive (**a**) and negative (**b**) variations of the Coddington shape factor before and after zeroization (z) using an ELP increment (ΔELP) on a non-uniform distribution. The maximum values obtained for each parameter are shown on the graph (Diopters, left ordinate axis). The value of the ELP increment used for zeroization is plotted against the CF value (in mm, right ordinate axis).

Figure 4 represents the variations of the Mean PE, SD, MAE, and RMS of the non-uniform distribution before and after optimization using a constant increment (Delta ELP). These values are comparable to those obtained with the uniform distribution; however, the increment in ELP required to zero out the mean PE is slightly lower in magnitude. 

### 3.3. Comparing Symmetrical and Existing Optical Designs

We also explored the refractive impact of the change in symmetrical designs with three realistic IOL designs: the Envista MX 60 (Bausch & Lomb, Rochester, NY, USA), the Acrysof SA60AT (Alcon, Fort Worth, TX, USA), and the Finevision Micro F (BVI-PhysIOL, Liège, Belgium). The postoperative refractive variations induced by these IOLs were calculated as if they replaced symmetrical IOLs of the same powers, refractive index, and central thickness in emmetropic eyes. 

**Figure 5:**
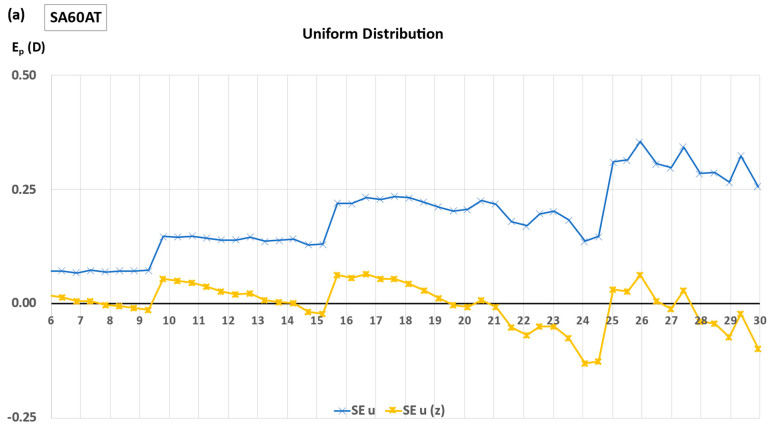

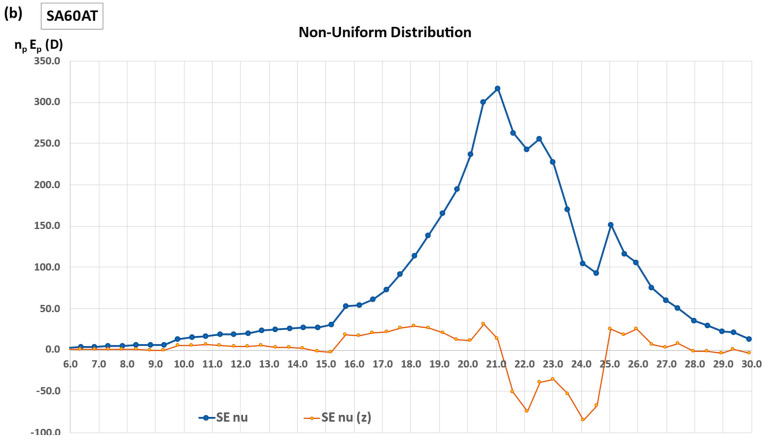

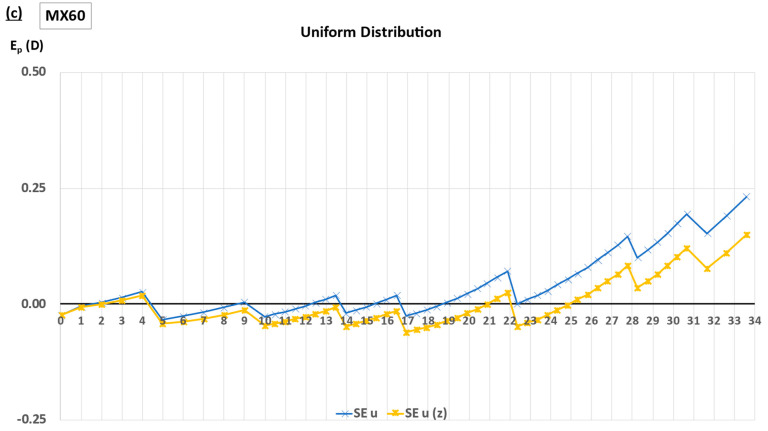

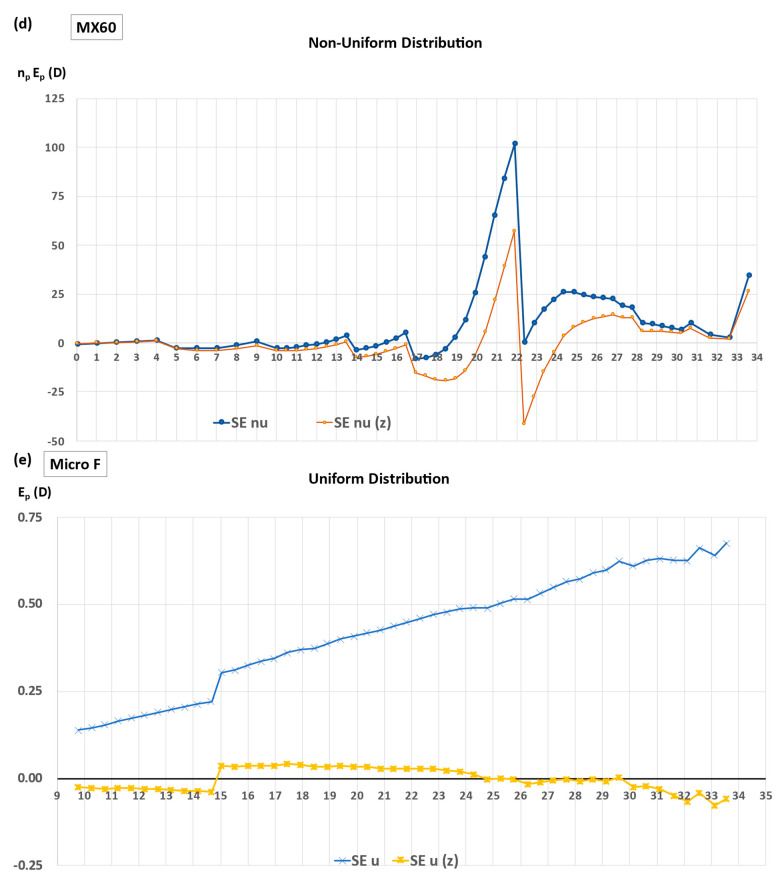

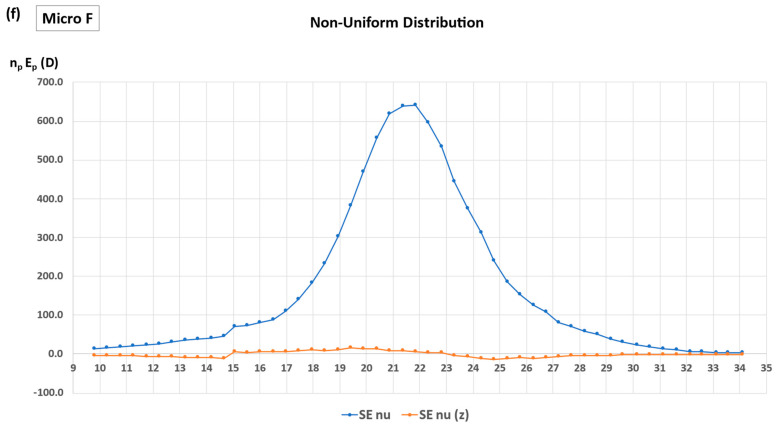
Spherical equivalent (uniform distribution, n_p_ = 1) and cumulative SE (non-uniform distribution) as a function of IOL power before and after zeroization for real IOL design. (**a**,**b**): SA60AT, (**c**,**d**): MX60, (**e**,**f**): Micro F.

Figure 5 shows the refractive impact of the design change from a symmetrical IOL to each of the three existing IOLs as a function of IOL power before and after zeroization for the uniform and non-uniform distributions.

**Figure 6:**
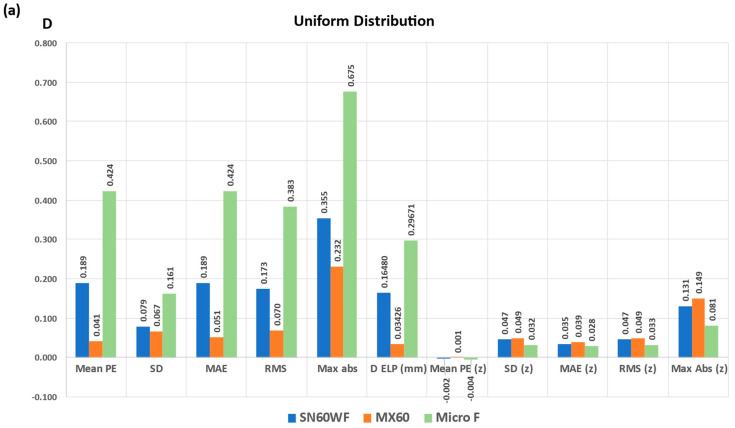

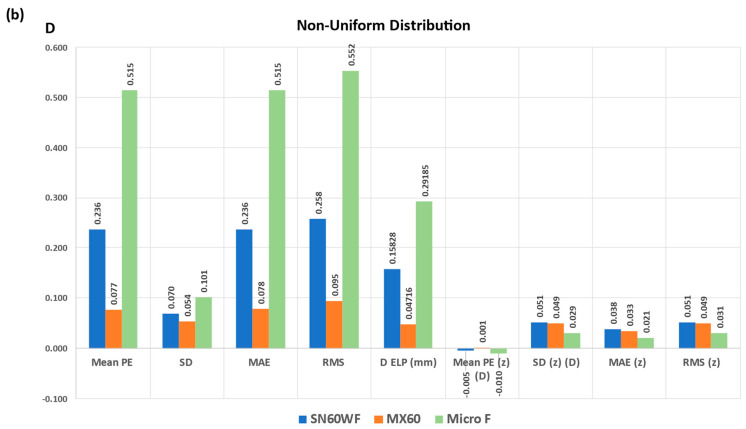
Mean prediction error (Mean PE), Standard Deviation (SD), Mean Absolute Error (MAE), and Root Mean Square (RMS) Maximum Absolute Error (Max Abs) of the PE for variations incurred by real IOL designs before and after zeroization (using a specific ΔELP increment) on the uniform (**a**) and non-uniform (**b**) distributions.

Figure 6 shows the numerical values before and after zeroization of Mean prediction error (Mean PE), Standard Deviation (SD), Mean Absolute Error (MAE), Root Mean Square (RMS), and Maximum Absolute Error (Max Abs) of the PE caused by the departure of real IOLs (SA60AT, MX60, and Micro F) from the symmetrical IOL design.

Before zeroization, the largest induced Mean PE, SD, RMS, and Maximum absolute error were obtained with the Micro F. After zeroization, these values reduced dramatically for all IOL models and were the lowest for the Micro F. 

## 4. Discussion

For the same dioptric power, the effective power of an intraocular lens placed at the same anatomical distance from the corneal apex varies with its design, i.e., the ratio between the dioptric powers of the back and front faces. We studied the theoretical effects of variations in IOL optical designs on the prediction error of a formula previously optimized for a symmetrical design with a uniform or realistic distribution of IOLs powers. While our work was limited to studying the theoretical effects of optical design variations on the error in an IOL power calculation formula, the sources of error in the refractive outcomes of cataract surgery are multiple [4,10]. In our model, freezing all variables but the Coddington shape factor could make it easier to analyze and interpret its influence on the SE regarding variance and bias. This approach can be helpful when dealing with complex models, such as thick-lens pseudophakic eyes, that include many variables and interactions between them. By simplifying the model in this way, we aimed to isolate the impact of the optical design of the IOL. We believe that understanding the impact of individual factors can provide interesting insights before introducing additional complexity. It is evident that in clinical practice, the impact of the studied variables may be diluted among those that influence the refractive outcome, but our theoretical model aims precisely to isolate those related to the design of the optics under paraxial conditions, and evaluate the effect of optimization.

Our results are, however, influenced by the choices made for the initial conditions, particularly the arbitrary choice of a symmetrical IOL design as a starting point. The interpretation of these results should consider the choice of a ‘neutral’ design. In practice, the variations from the design for which a formula is optimized would be more important to consider than the initial design. Using a uniform distribution to evaluate some precision metrics may seem unrealistic. However, it allowed better visualization of implant power’s impact on SE variations caused by design variations before and after zeroing out the mean PE.

Although theoretical, our results may help to appreciate the specific impact of the variations in optical design of a thick lens on the precision and accuracy of an IOL power formula. Most previous studies have highlighted the impact of implant power on refraction for the same deviation of the effective lens position [5,6]. IOL types are usually grouped according to similar designs for optimizing IOL power calculation [7,11]. The outcomes of a Monte-Carlo simulation utilizing a comprehensive biometric data set from a contemporary optical biometer were recently presented [12]. The simulation employed linear Gaussian optics approaches to examine the impact on spherical equivalent refraction when representing the IOL as a thick lens rather than a thin one. Adopting a more accurate thick lens model for the IOL (with a Coddington factor between −1.0 and 1.0) instead of a thin lens model (maintaining the same IOL power and axial position) led to a variation of up to ±1.5 diopters in spectacle refraction. These results are entirely consistent with those obtained from our model before optimization (Figure 1). In our simulations, the computed values of SD, MAE, and RMS do not appear to be very different depending on the type of distribution (uniform vs. non-uniform). The mean error must be zeroed out in clinical practice while adjusting the axial lens position. When the design variation implies a SE of the same sign for all IOL powers, zeroization necessarily induces the sign change for part of these SE powers to obtain a null mean PE. In all our simulations, the zeroization of the specific contribution of the optical design changes to the mean PE made it possible to considerably reduce the values of the indices usually used to quantify the precision of a formula 11 (SD, MAE, RMS). Our results show that the impact of optical design variations on the formula precision is less clinically significant after zeroization. In simulations performed using existing IOL designs, the largest SD was 0.051 D, and the MAE was 0.038D (Figure 5, non uniform distribution). The largest maximum absolute error went from 0.675 D before to 0.149 D after zeroization. These values are significantly lower than those usually measured when evaluating implant calculation formulas on large series of cataract-operated eyes. Langenbucher et al. conducted a study to evaluate the precision of different intraocular lens (IOL) power formulas following optimization on a training dataset [13]. Their research was conducted on a test set of 435 eyes and included formulas that utilized a single constant. The study found that the Haigis formula yielded the lowest mean absolute error (MAE) of 0.491D, while the Hoffer-Q formula resulted in the highest MAE of 0.546D. The largest maximum absolute error (2.756 D) was obtained with the Holladay formula. In their study, Melles et al. evaluated the precision of seven formulas using two extensive databases [14]. The BUII (±0.404 D) and Olsen (±0.424 D) formulas showed the least amount of variation, as evidenced by their lower standard deviation (SD), while the Hoffer Q formula (±0.473 D) demonstrated the highest amount of variation. Debellemanière et al. [9] conducted a comparison of 16 formulas using two groups of eyes consisting of 677 and 262 eyes, respectively. In the first group, the PEARL-DGS (±0.382 D), K6 (±0.394 D), Olsen (±0.394 D), EVO 2.0 (±0.398 D), RBF 3.0 (±0.402 D), and BUII (±0.402 D) formulas exhibited the least amount of variability, as indicated by their lower standard deviations (SDs). The accuracy in IOL power calculation of 24 traditional and modern vergence-based formulas, ray-tracing–based formulas, and artificial intelligence–based methods was recently investigated [15]. The MAE was comprised between 0.213 D (Castrop) and 0.583 D (Holladay 1) The formulas with the highest accuracy for the entire axial length group were VRF-G (SD ± 0.387 D), Kane (SD ± 0.395 D), Hoffer QST (SD ± 0.404 D), BUII (SD ± 0.405 D), RBF 3.0 (SD ± 0.408 D), Karmona (SD ± 0.409 D), PEARL-DGS (SD ± 0.412 D), and K6 (SD ± 0.416 D), based on the standard deviation values. 

The values obtained for these parameters in our simulation after optimization are lower by about an order of magnitude. These findings imply that the specific effects of IOL optical design variations observed in clinical settings are effectively counterbalanced by adjusting the constant to nullify the average prediction error. Furthermore, these variations may constitute a relatively minor component of the prediction error in implant calculation formulas once optimized.. When the Coddington factor is changed from null to positive values, the ELP value decreases because the main object plane of the implant is moved to its anterior surface [5]. This displacement causes a myopic refractive error. Inverse variations are observed when the Coddington factor changes towards negative values. The zeroization of the SE variation is obtained through a reduction of the effective lens position. Different strategies for formula constant optimizations exist [16,17], and our analysis was limited to a single-constant optimization. In clinical practice, many variables influence the postoperative refraction of a pseudophakic eye [4,10] and, therefore, the calculation of the optimal value of the constant. Depending on the cumulative influence of these variables, compensation for IOL design variations may vary.

The theoretical contribution of the change in optical design on the theoretical refractive accuracy of a formula appears to be proportional to its magnitude. Several lens models are widely recognized to maintain the shape of one or both IOL surfaces across a range of lens powers. The second surface is utilized to achieve the labeled equivalent power. As a result, one surface may exhibit only a limited variation throughout the entire delivery range. In contrast, the second surface is responsible for necessary adjustments to attain the designated equivalent power. These variations do not affect the value of the IOL power computed with Equation (A3), but modulate the lens’ “refractive effect” in the eye. Such effect is observed with the SA60AT, and MX60 designs and explains the sawtooth aspects observed in Figure 5a,b. Conversely, the variations in curvature occur simultaneously on the anterior and posterior surfaces for the Micro F. This may partly explain why the standard deviation is less after zeroization than with the MX60 and SA60AT, as this design is closer to the symmetrical one that we chose for the baseline. 

We restricted our analysis to biconvex-positive IOLs. Low or negative-power lenses can have a convex front and concave back (X > 1) [18]. In addition, manufacturers can produce IOLs in a manner that results in differences between the haptic plane and ELP across various IOL powers; this represents a potential additional source of variability that we have not analyzed.

We used identical corneal power in all our simulations to isolate the impact of the optical design on postoperative refraction. Considering several corneal powers and eyes modeled with various values of effective implant positions would certainly impact the numerical values reported. We believe that these results are however generalizable because we have previously shown the relatively weak impact of corneal power in the refractive variations of pseudophakic eyes caused by changes in ELP and/or design [6,8]. Nevertheless, the observed trends are robust to the numerical parameters’ value. Interestingly, the values obtained with the uniform distribution are relatively close to those obtained with the unimodal distribution measured in our population. This trend is certainly related to these two distributions centered on fairly similar IOL power mean values.

A limitation that hinders further improvement in calculating the power of intraocular lenses (IOLs) is the reliance on the thin lens model, which becomes problematic in short eyes requiring high-power IOLs [3,10,19]. The central thickness of the IOL cannot be overlooked [20], requiring the consideration of additional factors such as the optical design (Coddington shape factor, central thickness, and refractive index). However, our results suggest that the gain expected by considering optical design alone will remain modest if the formula is well optimized. 

Our study was limited to the distribution ratio variation between the implants’ front and back. The geometry of the different implant models also involves the position and angulation of the haptics, the refractive index, and the optic thickness. In a paraxial model of thick lenses, these parameters are responsible for the positions of the principal planes of the implant. Refraction is also influenced by non-paraxial variables such as optical asphericity [21], but our simulations were restricted to paraxial considerations. 

## 5. Conclusions

In conclusion, changes in the Coddington factor, which describes the bending of the lens, can occur significantly over the delivery range and affect the accuracy of IOL power formulas. It may be possible to enhance the accuracy of IOL power prediction formulas and IOL constant optimization by considering the shape, thickness, and refractive index of the IOL material and the IOL power. Zeroization is an important step to reduce the impact of optical design changes.

## Figures and Tables

**Table 1:** Biometric and symmetrical IOL-related variables values used for numerical simulations. All distances are in mm.

Power (D)	Thickness	Ria = −Rip	S1S3	Axial Length	Power (D)	Thickness	Ria = −Rip	S1S3	Axial Length
0.5	0.38	655.979	6.5	30.79	18	0.851	18.176	5.1	24.55
1	0.392	327.979	6.46	30.58	18.5	0.866	17.682	5.06	24.40
1.5	0.404	218.645	6.42	30.37	19	0.881	17.215	5.02	24.25
2	0.416	163.977	6.38	30.16	19.5	0.896	16.771	4.98	24.11
2.5	0.428	131.177	6.34	29.95	20	0.911	16.350	4.94	23.96
3	0.44	109.309	6.3	29.74	20.5	0.929	15.949	4.9	23.82
3.5	0.452	93.690	6.26	29.54	21	0.947	15.567	4.86	23.68
4	0.464	81.975	6.22	29.34	21.5	0.965	15.203	4.82	23.54
4.5	0.476	72.863	6.18	29.14	22	0.983	14.855	4.78	23.41
5	0.488	65.573	6.14	28.95	22.5	1.001	14.523	4.74	23.27
5.5	0.5	59.609	6.1	28.76	23	1.019	14.205	4.7	23.14
6	0.512	54.639	6.06	28.56	23.5	1.037	13.901	4.66	23.00
6.5	0.524	50.433	6.02	28.38	24	1.055	13.609	4.62	22.87
7	0.536	46.828	5.98	28.19	24.5	1.073	13.329	4.58	22.74
7.5	0.548	43.703	5.94	28.00	25	1.091	13.060	4.54	22.61
8	0.56	40.969	5.9	27.82	25.5	1.109	12.802	4.5	22.48
8.5	0.572	38.557	5.86	27.64	26	1.127	12.553	4.46	22.35
9	0.584	36.412	5.82	27.46	26.5	1.145	12.314	4.42	22.22
9.5	0.596	34.494	5.78	27.28	27	1.163	12.084	4.38	22.10
10	0.611	32.767	5.74	27.11	27.5	1.184	11.862	4.34	21.97
10.5	0.626	31.204	5.7	26.94	28	1.205	11.648	4.3	21.85
11	0.641	29.783	5.66	26.77	28.5	1.226	11.441	4.26	21.73
11.5	0.656	28.486	5.62	26.60	29	1.247	11.242	4.22	21.61
12	0.671	27.297	5.58	26.43	29.5	1.268	11.049	4.18	21.49
12.5	0.686	26.202	5.54	26.26	30	1.289	10.862	4.14	21.37
13	0.701	25.192	5.5	26.10	30.5	1.31	10.682	4.1	21.25
13.5	0.716	24.257	5.46	25.94	31	1.331	10.507	4.06	21.13
14	0.731	23.389	5.42	25.78	31.5	1.352	10.338	4.02	21.02
14.5	0.746	22.580	5.38	25.62	32	1.373	10.174	3.98	20.90
15	0.761	21.825	5.34	25.46	32.5	1.394	10.016	3.94	20.79
15.5	0.776	21.119	5.3	25.30	33	1.415	9.861	3.9	20.67
16	0.791	20.457	5.26	25.15	33.5	1.436	9.712	3.86	20.56
16.5	0.806	19.835	5.22	25.00	34	1.457	9.567	3.82	20.45
17	0.821	19.249	5.18	24.84	34.5	1.478	9.426	3.78	20.34
17.5	0.836	18.697	5.14	24.69	35	1.499	9.289	3.74	20.23

**Figure A1:**
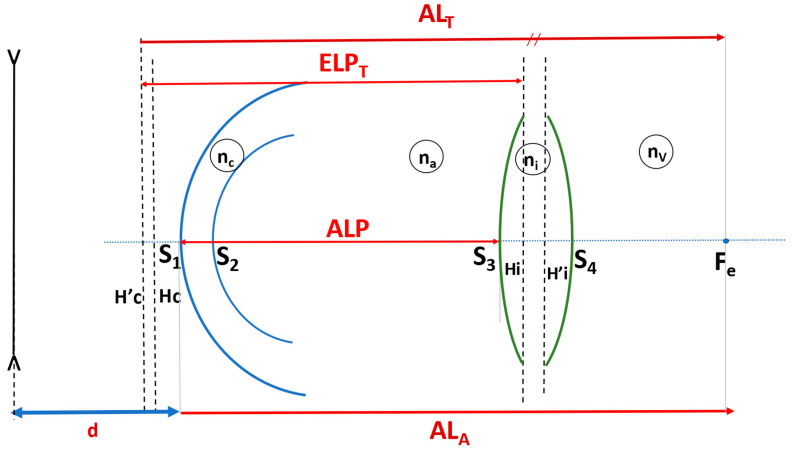
Paraxial, four-surface, thick-lens, pseudophakic eye model. F′_e_ is the back focal point of the paraxial schematic pseudophakic eye. AL_A_ is the anatomic axial length from the anterior surface of the cornea to the photoreceptor plane (PP). It is equal to the distance S_1_F′_e_ if the eye is emmetropic. The AL_T_ is the thick lens axial length, which connects the principal image plane of the cornea H′_c_ to the PP and is reduced by the distance H_i_H′_i_ separating the two principal planes of the IOL. ALP is the anterior lens position connecting the corneal vertex (S_1_) to the IOL vertex (S_3_); ELP_T_ is the effective thick lens position joining the principal image plane of the cornea to the principal object plane of the IOL; and d is the distance to the spectacle plane.

**Figure A2:**
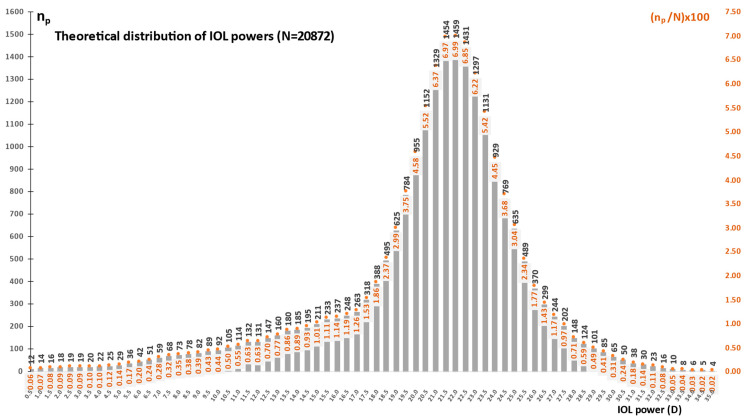
Theoretical distribution of the emmetropizing IOL powers in a population of 20872 eyes of cataract surgery candidates without previous ocular surgery.

## Data Availability

Data available on request.

## Appendix A

Figure A1 depicts a four refractive surface pseudophakic eye.

The postoperative refraction of the modeled pseudophakic eye is given by [8]:

(A1) $$R=-\frac{1}{\frac{1}{\frac{n_{a}}{\frac{n_{a}}{-\frac{n_{v}}{\left({AL}_{T}-{ELP}_{T}\right)}+P}-\left({ELP}_{T}\right)}+K}-d}$$

where:

- K is the total power of the cornea computed with the following formula:

(A2)
$$K=\frac{n_{s}-1}{R_{ca}}+\frac{n_{a}-n_{s}}{R_{cp}}-\frac{\left(n_{s}-1\right)\left(n_{a}-n_{s}\right)d_{c}}{n_{s}R_{ca}R_{cp}}$$

R_ca_: radius of curvature of the anterior cornea, R_cp_: radius of curvature of the posterior cornea, n_s_: refractive index of stroma, n_a_ refractive index of aqueous, d_c_: corneal thickness.

- P is the total power of the IOL given by:

(A3)
$$P=\frac{n_{i}-n_{ss}}{R_{ia}}+\frac{n_{ss}-n_{i}}{R_{ip}}-\frac{\left(n_{i}-n_{ss}\right)\left(n_{ss}-n_{i}\right)d_{i}}{n_{i}R_{ia}R_{ip}}$$

R_ia_: radius of curvature of the anterior surface, R_ip_: radius of curvature of the posterior surface, n_i_: refractive index of the IOL, n_a_: refractive index of aqueous, d_i_: IOL thickness.

- ELP_T_ is the distance between the principal image plane of the cornea and the principal object plane of the IOL. Its relationship with the anatomical lens position of the IOL (S_1_S_3_ = ALP) is expressed as follows:

(A4)
$${ELP}_{T}=\bar{S_{1}S_{3}}+\bar{{H^{\prime }}_{c}S_{1}}+\bar{S_{3}H_{i}}$$

- AL_T_ joins the anterior surface of the cornea to the photoreceptors’ plane at the fovea.

The relationship between the anatomical axial length and LT is given by:

(A5) $$AL_{T}={AL}_{A}-\bar{S_{1}{H^{\prime }}_{c}}-\bar{H_{i}{H^{\prime }}_{i}}$$

For the same ALP, optical power, and central thickness, a variation in the shape factor results in an axial displacement of the principal planes of the IOL. Therefore, the design of a thick lens IOL influences the value of the ELP_T_ via the position of the main plane image S_3_H_i_ and the value of L_T_ via the H_i_H’_i_ segment.

The distance S_3_H_i_ is given by:

(A6) $$S_{3}H_{i}=\frac{n_{a}\left(n_{v}-n_{i}\right)d_{i}}{{n_{i}R}_{ip}D_{i}}$$

The distance S_4_H’_i_ is given by:

(A7) $$S_{4}{H^{\prime }}_{i}=-\frac{n_{v}\left(n_{i}-n_{a}\right)d_{i}}{{n_{i}R}_{ia}D_{i}}$$

The segment H_i_H’_i_ is given by:

(A8) $$\bar{H_{1}{H^{\prime }}_{i}}=\bar{S_{3}S_{4}}-\bar{S_{3}H_{i}}-\bar{S_{4}{H^{\prime }}_{i}}$$

The refractive consequences of IOL design variations can be calculated using Equation (A1) that provides the refraction of a pseudophakic eye based on various biometric variables, including ELP_T_ and AL_T_.

## Appendix B

Figure A2 plots the theoretical distribution of IOL powers to induce emmetropia, computed with the PEARL DGS formula, in a population of 20872 eyes.
